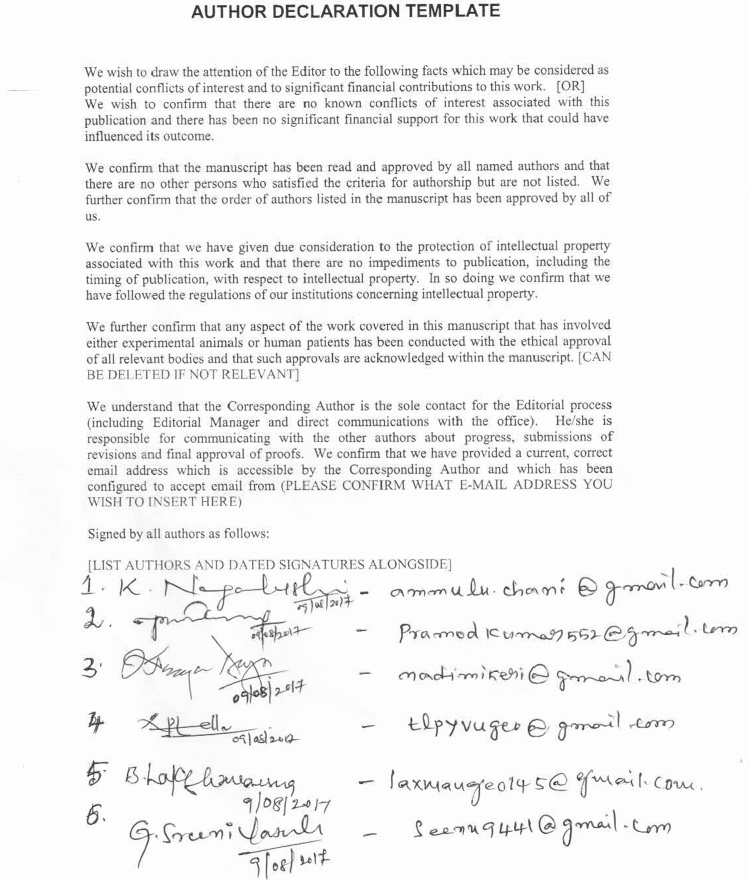# Dynamics of Pulicat Lake mouth analysis using geospatial data, east coast of India: Implications to socio-economic scenarios

**DOI:** 10.1016/j.dib.2017.09.016

**Published:** 2017-09-15

**Authors:** K. Nagalaksmi, Madri Pramod Kumar, N. Jayaraju, T. Lakshmi Prasad, B. Lakshmanna, G. Sreenivasulu

**Affiliations:** a Department of Earth Sciences, Yogi Vemana University, Kadapa 516003, India; b Department of Geology, Yogi Vemana University, Kadapa 516003, India

**Keywords:** Pulicat Lake, IRS LISS III data, Multi-temporal satellite imageries, Socio economic settings

## Abstract

Pulicat Lake is one of the major wetlands in India. It is the second largest brackish water lagoon in India next to Chilika Lake in Orissa state. Pulicat Lake sits beside the Bay of Bengal so, the study on the mouth is vital. The investigations were carried out by using multi-temporal satellite imageries of IRS P6, LISS III data for four years viz., 2009, 2011, 2012 and 2013. Subsequent changes in the width of the lake at the southern side were measured. It is found that the lake mouth is not static but dynamic predominantly fluctuating year by year. Obviously, this poses threat to the lake biodiversity. Hence, it is high time to mitigate, manage, monitor and protect the existing width of the sea mouth to keep the lake biological, ecological, economically active. This paper noticed a considerable change in the mouth of the lake studied using satellite imageries and socio-economic settings

**Specifications Table**

**Table:** 

Subject area	*Earth and Planetary Sciences*
More specific subject area	*Remote Sensing*
Type of data	*Table, figures*
How data was acquired	*IRS6, LISS III Satellite Imageries*
Data format	*Analyzed*
Experimental factors	*Satellite imageries were georeferenced by using GIS & ERADAS imagine software*
Experimental features	*River mouth dynamics were calculated for the period 2009–2013*
Data source location	*Pulicat Lake, Nellore district, Andhra Pradesh, India*
Data accessibility	*The data are available with this article*

**Value of the data**

• It can serve as baseline data for the lake mouth interpretation.
• Data presented here can be used to understand lake mouth dynamics between the period 2009–2013.
• Data are georeferenced and it can be utilized in future studies.
• It is also useful to researchers, stakeholders, policymakers, working on lake ecosystem.
• It is useful for the socio-economic settings of fisherman sustained by the lake.

## 1 Data

**Fig. 1:**
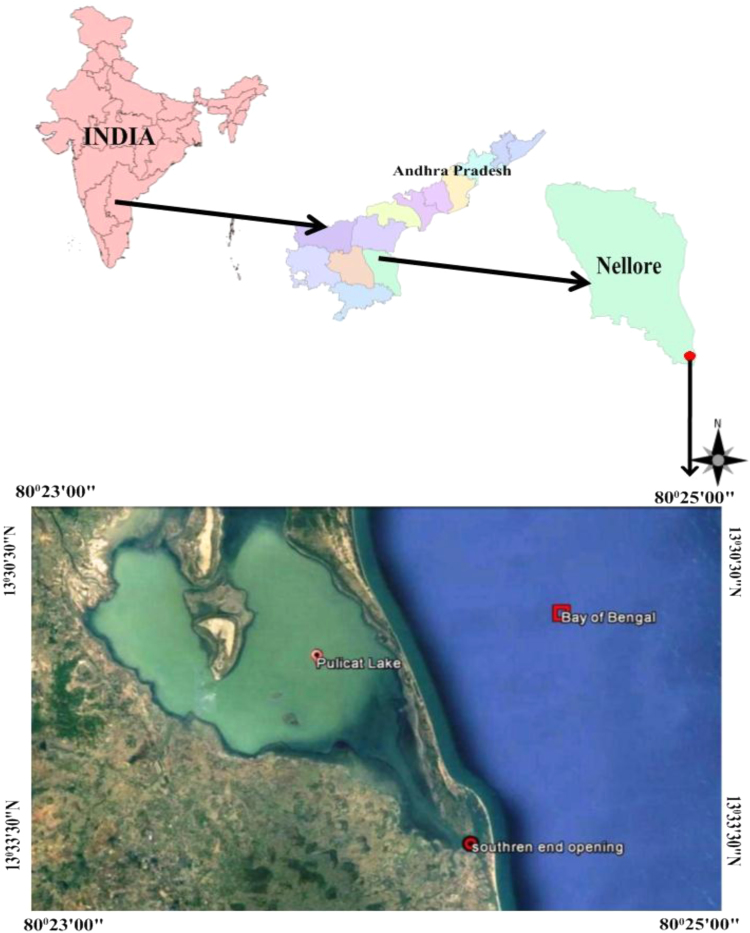
Location map of the study area.

**Fig. 2:**
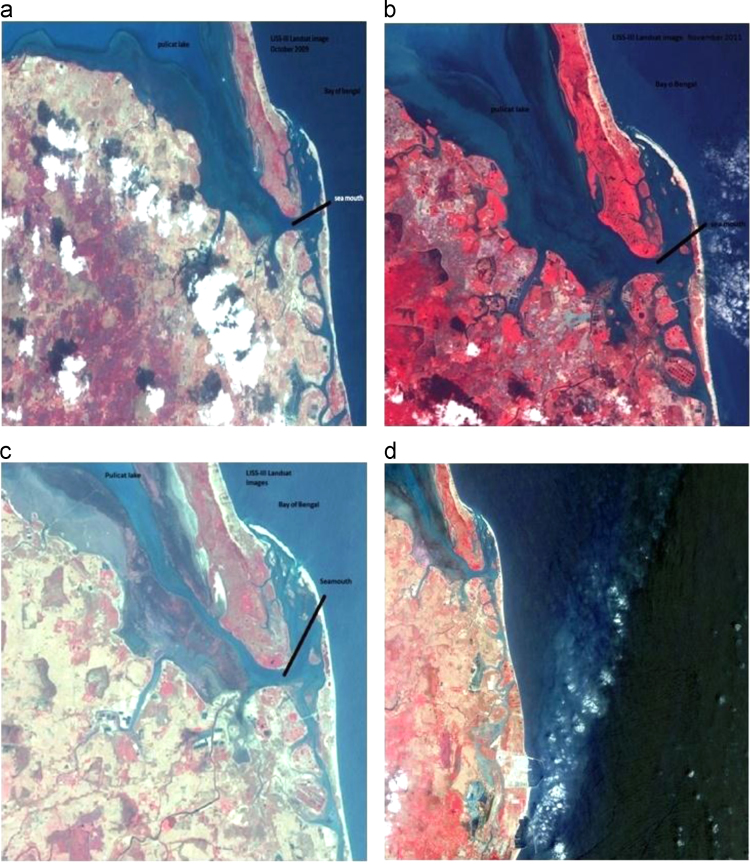
Satellite imageries showing southern part of the mouth opening dynamics in different periods. (2a) October 2009 LISS III Image, (2b) November 2011 LISS III Image (2c) March 2012 LISS III image, (2d) March 2013 LISS III image.

The data discloses the fluctuations of Pulicat lake mouth width for four years viz., 2009, 2011, 2012 and 2013, by using satellite imageries and ground truths. The salient features of the satellite imageries and appraises of mouth dynamics are shown in Table 1, Table 2. The location map of the investigation area and the satellite imageries showing variations in mouth opening in different time periods are shown in Fig. 1, Fig. 2.

**Table 1:** Salient features of Satellite data sets.

**Name of the Satellite**	**Date of data acquisition**	**Sensor**	**Resolution**	**Path**	**Row**	**No. of bands**
IRS- P6 (Resourcesat-2)	24th October 2009	LISS III	24 m	102	064	4
IRS- P6 (Resourcesat-2)	19th November 2011	LISS III	24 m	102	064	4
IRS- P6 (Resourcesat-2)	18th March 2012	LISS III	24 m	102	064	4
IRS- P6 (Resourcesat-2)	13th March 2013	LISS-III	24 m	102	064	4

**Table 2:** Pulicat Lake mouth width on the southern part for the Years 2009, 2011, 2012 and 2013.

**Year**	**Sea mouth width in meters (Measured through satellite imageries)**	**Sea mouth width in meter (Field observations)**
2009 October	548 m	549 m
2011 November	558 m	560 m
2012 March	508 m	507 m
2013 March	544 m	543 m

## 2 Experimental design, materials and methods

### 2.1 Importance of data

The lagoon has two openings into the Bay of Bengal one at northern side and another at the south end. The northern opening is filled up by the sediments due to accretion processes [1]. From the satellite imageries, it is observed that a spiti- like structure is springing up at the southern end. It is important to know the erosion or accretion phase of the estuary mouth because estuaries are ideal habitats for diverse biota by virtue of mixing of fresh and sea water. If the southern opening also filled up with sediments (closed by sediments) leads to the ecological imbalances, and deprives the sustenance of 34 village people who are dependent on the lake for their livelihood. In this backdrop, the present study has been initiated in the larger interest of human settlements with reference to the biodiversity

### 2.2 Methodology

The study has made use of various datasets, includes Survey of India (SOI) toposheets of 1:50,000 scale, IRS LISS – III geo-coded data of 1:50,000 imageries and field measurements. Firstly, a baseline map was prepared by using toposheets 66C/1. The Satellite datasets were downloaded from National Remote Sensing Centre (NRSC) handling Bhuvan website for the periods October 2009, November 2011, March 2012 and March 2013. The imageries were cropped to the Area of Interest (AOI) and processed for geometric corrections by using EARDAS software. The cropped and processed datasets were georeferenced by projecting the imageries to the standard projection i.e., UTM _WGS 1984. Then processed satellite imageries were interpreted visually and digitally by using the image interpretation elements such as tone, texture, shape, pattern, association etc., in ArcGIS 9.3. The results obtained from the Remote Sensing and Geographic Information system technique were cross checked by the ground truth including field measurements.

### 2.3 Geospatial analysis of Lake Mouth dynamics

Pulicat Lake is basically a shallow lagoon, whose average depth about 1.5 m at the beginning of the 20th century, has shrunk to less than one meter now owing to siltation of the lagoon [2]. Changes in the lake mouth into the Bay of Bengal were determined or quantified by measuring the width of the opening by using satellite imageries. Remotely sensed multi- temporal data acquired for four years from 2009 to 2013 reveal that the lake mouth and the formation of spiti (sand bar) – like structure is highly dynamic. In the present study, the width of the opening point in the year 2009 is taken as reference, and measured the width of the estuary mouth for succeeding years with respect to it. From the satellite imageries it is observed that the width of the mouth in October 2009 was about 548 m (Fig. 2a), and was increased by 10 m i.e., 558 m in November 2011 (Fig. 2b), after that width of mouth began to shrink and decreased from 558 m to 508 m in the year March 2012. Almost 50 m of width got reduced, due to immense sedimentation (Fig. 2c) and in 2013 October it is observed that the width of the mouth is 544 m (Fig. 2d). The measurements obtained by using satellite imageries were cross-checked by the field measurements, more or less coincide with the former values (Table 2).

Coastal environments and estuaries are products of interaction between marine, physical, meteorological and biological activities with the geology and sediments [3]. Coastal dynamics by and large controlled by the waves, currents and tide activities. Measurements from both satellite imageries and field observations show that the mouth opening on the southern side was alternatively increased and decreased year by year in the period of study. It is inferred that abrupt decline in the width of the mouth opening in the year 2012 is due to prevailing low atmospheric pressure conditions, low wave and current activities responsible for accretion of sediments towards the southern side (Fig. 2c). It clearly depicts the huge deposition of sediments. Enormous Mangroves stilt like roots catches silt (mud deposits) at the mouths of stream that further slow down the waves and currents thus allows the sediments to settle down gradually.

## Figures and Tables

**Figure:**